# Effects of immersive virtual nature on nature connectedness: A systematic review and meta-analysis

**DOI:** 10.1177/20552076241234639

**Published:** 2024-03-25

**Authors:** Elena Brambilla, Evi Petersen, Karen Stendal, Vibeke Sundling, Tadhg E MacIntyre, Giovanna Calogiuri

**Affiliations:** 1 Centre for Health and Technology, Department of Nursing and Health Sciences, University of South-Eastern Norway, Drammen, Norway; 2 Department of Sports, Physical Education and Outdoor Studies, University of South-Eastern Norway, Bø, Norway; 3 Department of Early Childhood Education, Oslo Metropolitan University, Oslo, Norway; 4 Department of Business, Marketing and Law, University of South-Eastern Norway, Ringerike, Norway; 5 Department of Optometry, Radiography and Lighting Design, University of South-Eastern Norway, Kongsberg, Norway; 6 All Institute, Department of Psychology, 8798 Maynooth University, Maynooth, Irlanda; 7 Section for Public Health, Department of Public Health and Sport Sciences, Inland Norway University of Applied Science, Elverum, Norway

**Keywords:** Nature connectedness, nature exposure, nature-based interventions, immersive virtual environment, virtual reality, technological nature, virtual nature

## Abstract

**Objective:**

This study systematically summarizes the extant literature on the impacts of immersive virtual nature (IVN) on nature connectedness in the general population.

**Methods:**

Papers were considered eligible if peer-reviewed, in English language, comprising experimental or quasi-experimental trials, including at least one outcome relative to nature connectedness in the general population. Database search was conducted on Scopus, Web of Science, Google Scholar, Medline, and GreenFILE (22–28 November 2021). Risk of bias was established by the Cochrane RoB 2 tool. Data synthesis was conducted through meta-analysis according with the Cochrane Consumers and Communication Group guidelines.

**Results:**

Six eligible papers (9 studies; n = 730) were selected, in which IVN was compared to *(i)* non-immersive virtual nature, *(ii)* immersive virtual built environments, *(iii)* non-immersive virtual built environments, and *(iv)* actual nature. The risk of bias was predominantly “low” or of “some concerns.” Meta-analyses showed a statistically significant overall effect for the first (*g* = 0.26; *95% CI* = 0.06–0.45; *I^2^* = 35%) and fourth group (*g* = −1.98; *95% CI *= −3.21 to −0.75; *I^2^* = 96%), the former in favor of IVN and the latter in favor of actual nature. Subgroup analyses were conducted for the first and second groups of studies to explore possible sources of heterogeneity. The small number of studies available limits the validity of the outcomes of the meta-analyses.

**Conclusion:**

The findings indicate that IVN may be an effective tool for the promotion of nature connectedness, although the evidence in this field is still limited and largely mixed. Recommendations for future research are discussed.

## Introduction

### Nature connectedness and wellbeing

Accumulating evidence demonstrates the beneficial effects on physical and mental health derived from human–nature interactions. Physical interactions with nature, such as viewing natural landscapes or visiting naturalistic locations are positively associated with wellbeing and have been acknowledged as a significant health-promoting factor among different societal groups.^1,2^ In this respect, the concept of nature connectedness has gained increasing attention, as conveyed by a 72.6% increase of papers on this subject from 2010 onwards.^3^ Nature connectedness includes a range of competing conceptualizations describing a person's relationship and emotional attachment to the natural world, as well as the beliefs and behaviors associated with it. This construct may be also seen as going beyond a general affinity with nature and rather conceptualizing nature as part of an individual's identity.^4^

The importance of the role of nature connectedness for people's health is highlighted by its association with several indicators of social, physical, and psychological wellbeing,^5–7^ such as happiness, mood, vitality, and satisfaction with life.^8–10^ Studies also reported that a higher sense of nature connectedness is associated with improved self-reported vitality and reduced symptoms of mental disorders associated with stress, regardless of cultural and climatic factors.^11–13^ Further, increased nature connectedness was found to mediate the positive affective responses associated with experiences in nature, as well as lead to increased perceived coping capability.^11^ It is also plausible that nature connectedness may support and enhance people's health through enhanced physical activity in natural settings and heightened motivation to visit natural environments, providing the conditions for people to gain the health benefits associated with an active lifestyle (a known important health-related behavior) and direct nature contact.^14^ In this respect, an integrative review indicated that having a higher sense of nature connectedness makes people more likely to view naturalistic locations (e.g. forests, mountains, open spaces, etc.) as attractive places for walking, playing, or exercising.^15^ Accordingly, the desire to experience nature was an important motive for engaging in physical activity, especially among those who tend to be physically active outdoors in nature rather than in gyms and sports facilities.^16,17^

### How to foster nature connectedness?

Nature connectedness is often viewed as an individual's relatively stable personality trait,^6^ which is primarily supported by frequent experiences of nature during childhood.^16,18,19^ However, accumulating evidence supports the idea that nature connectedness, at least to some extent, can be modulated by specific activities or circumstances. For instance, a recent meta-analysis found that a variety of direct (e.g. tours or guided mindfulness in natural settings) and indirect experiences of nature (e.g. guided imagery or mobile applications that encourage to notice nature) were effective in eliciting increased levels of nature connectedness in adults.^20^ Studies showed that nature connectedness can be influenced even by brief interactions with nature, such as walking in an arboretum for 10 min or planting trees.^11,21^ However, due to increased urbanization, opportunities for engagement in indoor activities are increasing while nature experiences are globally decreasing, with a consequent impact on the population's health.^22–24^ Urban nature's degraded status and the limited access to green spaces, for example, are associated with consistent negative health outcomes^22^ affecting satisfaction with life and happiness.^9,25^ Researchers have warned that this increasing distancing from the natural world may cause a general impoverishment of people's sense of nature connectedness, especially among children, with deleterious consequences for their health.^26,27^

### Immersive virtual nature

Prolonged screen-time such as playing videogames and watching television, has been suggested to exacerbate people's disconnection from nature.^24^ Conversely, technology provides alternative ways to engage with nature.^28,29^ Technological nature is a broad concept defined as “technologies that in various way mediate, augment or simulate the virtual world,”^30^ and it includes a variety of artifacts and digital technology.^29^ Among these, virtual reality (VR), a technology that provides a continuous stream of computer-generated input to create the illusory perception of being in a virtual world,^31^ can deliver highly realistic experiences of nature.^28^ This technology often relies on the use of head-mounted displays (HMDs), commonly called “VR masks” or “VR headsets.” The HMD blocks the users’ view of the physical surroundings while at the same time providing a 360° range of vision to a virtual scenario. HDMs and VR systems are growing in popularity and becoming increasingly affordable in the consumer market.^28,29^ However, the visual stimulation provided by many HMDs is not well tolerated by all users, and often causes so-called cybersickness, a particular form of motion sickness characterized by symptoms such as nausea, vertigo, and general malaise.^32^

Immersive virtual nature (IVN) specifically refers to a VR system that delivers an immersive view of a naturalistic scenario.^32^ To date, mostly two types of IVN scenarios exist, which are developed using two distinct techniques: 360° videos and computer-generated scenarios. 360° videos, are created using 360° cameras that allows to film, either statically (i.e. with a fixed viewpoint) or dynamically (i.e. with the viewpoint moving in the space), an existing environment from all angles simultaneously. While this technique usually provides highly realistic representations of the actual natural environment, it generally only allows for a passive view of the scenario from behalf of the user. Differently, computer-generated scenarios are developed through video game visualization techniques. They may appear artificial with respect to shapes, biodiversity, and colors of the natural elements, though recent advancements in photorealistic techniques allow for more detailed and credible representations of nature. On the other hand, these scenarios can provide the users with different levels of interactivity through hand-held controllers and tracking sensors placed on participants’ bodies and tracked by infrared cameras, though this varies largely depending on the design of the specific scenario.^33,34^

Since interactions with nature are fundamental to the development of nature connectedness,^35^ researchers have been investigating the potential of IVN to achieve similar outcomes. Since, compared to less immersive forms of technological nature (e.g. phone or television screens), IVNs can provide the users with a greater sense of presence (i.e. the extent to which a person feels like they *are* in a given virtual environment^36^), as well as psychological responses more similar to those associated with experiences in actual nature,^37^ this technology may also be more effective in eliciting increased feelings of nature connectedness. Even though acute changes in nature connectedness elicited by brief IVN exposures may only be temporary or short-lived, repeated exposures may allow for long-term and stable changes to occur.

### The current study

The interest in the effects of IVN on psychological outcomes has been rapidly evolving in the past years, with the number of studies in this field increasing considerably in recent times.^20,28,37,38^ For instance, the meta-analysis by Sheffield et al.^20^ examined the effects of various ways of interacting with nature (either directly or indirectly, including digitally-mediated interactions) on people's sense of nature connectedness, highlighting the potential of IVN among other digital and non-digital media.^20^ Further, narrative reviews highlighted the way in which engaging with IVNs may foster people's nature connectedness by boosting past nature experiences through savoring^28^ and enhancing people's motivation to visit naturalistic locations.^33^ Yet, to date, a review that systematically synthetizes the scientific evidence generated by experimental studies investigating the effects of IVN on nature connectedness outcomes is still missing. With the present study, we aim at addressing this gap. In particular, this review study relied on a broad conceptualization of nature connectedness, including evidence from experimental studies with samples derived from the general population.

The following research question underpins this systematic review:

What are the effects of IVN exposure on nature connectedness in the general population?

## Methods

A protocol for the review described in this article was registered ahead of conducting the search (PROSPERO: CRD42021290442) and published, previous peer-reviewing, as a journal publication.^39^ The present article is structured according with the Preferred Reporting Items for Systematic Reviews and Meta-Analyses (PRISMA) guidelines.^40^

### Eligibility criteria

The PICOS framework^41^ was employed to define specific inclusion and exclusion criteria to identify eligible studies (Table 1). Studies were included if available as peer-reviewed journal articles or post-review preprints reporting primary findings and written in the English language. To ensure higher quality of the evidence included, papers that did not undergo peer-review (e.g. reports, working papers, and pre-review preprints) and other gray-literature publications (e.g. theses and conference papers) were considered non-eligible. No restrictions were set with respect to geographical location and publication date.

**Table 1.** Eligibility criteria.

Parameter	Inclusion	Exclusion
Participants/population	General population (any age).	Clinical population, e.g. hospitalized patients and people following rehabilitation programs.
Intervention	Any IVN, conceptualized as digital simulations of natural scenarios (such as natural environment, wildlife, and fauna) through highly immersive (e.g. HMDs, CAVE, immersive room) and semi-immersive VR technology (e.g. immersive screen or display, mixed-reality devices).	Non-immersive VR (e.g. desktop, smartphone, pictures) or other forms of digital reproductions of nature.
Comparison	Any comparison that did not employ IVN Placebo or control conditions No comparison or control.	No exclusion criteria applied.
Outcome	Any quantitative outcome indicative of nature connectedness, including assessments through validated instruments, but also other measurement of individuals’ beliefs, attitudes, intentions, and behaviors relative to nature, natural environments, or the wildlife.	Qualitative assessments of NC.
Study design	Controlled experimental trials (randomized or non-randomized) and quasi-experimental studies with parallel groups and/or pre–post design Uncontrolled trials with pre–post assessments.	Correlational studies, qualitative studies with no quantitative evaluation, research protocols, and reviews of literature.

CAVE: Cave Automatic Virtual Environments; HMD: head-mounted displays; IVN: immersive virtual nature; VR: virtual reality.

### Participants/population

Consistently with the present study's aim and published protocol,^39^ the inclusion criteria covered any group or subgroup representative of the general population such as healthy adults, students, and participants in public events. No exclusion criteria were employed concerning participants’ age and previous experiences with VR technology. Studies that exclusively involved samples derived from clinical populations, such as specific groups of patients, were considered not-eligible. Examples included individuals diagnosed with Alzheimer's disease, cancer, stroke, paralysis, post-traumatic stress disorder, or depression. This choice was motivated by the understanding that clinical populations and specific patient groups warrant specific considerations, and the outcomes observed in samples exclusively composed of participants with these characteristics may not be generalizable to the general population.

### Intervention

Interventions involving exposure to an IVN through immersive or semi-immersive technology were considered eligible. For the scope of this study, IVNs had to display natural environments as defined by Calogiuri & Chroni's: “open outdoor spaces that allow the individual to be surrounded by the elements of nature (trees, plants, grass, mountains, water, etc.)”.^15^ In line with previous conceptualizations,^42,43^ immersion was operationalized as any device providing a 360° view of a virtual environment while impeding the vision to the external (actual) premises, hence creating the illusion of being within the virtual world. Examples of immersive technology include immersive rooms, HMDs, and Cave Automatic Virtual Environments. For the purpose of this study, interventions that exposed participants to IVN though semi-immersive technology were also considered eligible. This referred to devices that only partially exclude the view of the real environment or do not allow a 360° view on the virtual environment. Example of semi-immersive devices includes HMDs with range of vision below 360°, mixed-reality systems (i.e. devices that augment views on the real world with virtual elements), and immersive screens (i.e. projections on curved and surrounding surfaces). IVN developed as either 360° videos or computer-generated scenarios were considered eligible. Exposure could be delivered as either passive contemplation of the IVN (e.g. observing the virtual environment in a seated position, without any interaction with it), or an interactive experience (e.g. picking objects or navigating within the virtual scenario). Interventions that simulated nature exclusively through non-immersive technologies (e.g. pictures, smartphones, television, or computer screens) were considered not-eligible.

### Comparison

No limitations to eligibility were set with respect to the characteristics of the comparison. Studies were considered eligible if they involved any comparison that did not employ IVN, including exposure to non-immersive virtual nature, placebo, or control conditions. Studies with no comparison or control were also considered eligible, assuming that they included pre-post assessments.

### Outcomes

Eligible outcomes included any measurement indicative of an individual's sense of nature connectedness. This comprised assessments through established instruments (e.g. the *Inclusion of Nature in Self Scale,*^44^ the *Connectedness to Nature Scales*,^6^ and the *Nature Relatedness Scale*^45^), but also any other psychological measurements such as beliefs, attitudes, intentions, and behaviors relative to nature, natural environments, or the wildlife (e.g. affective beliefs about nature, attitudes toward wildlife, or intention to visit naturalistic locations) assessed through validated or not validated instruments.

### Study design

Randomized and non-randomized controlled trials (RCTs; either with parallel conditions or crossover design), with pre–post or only post-assessments, and uncontrolled trials with pre–post assessments were considered eligible. Correlational studies were excluded.

### Information sources

Relevant papers were systematically searched in five databases: Web of Science, GreenFILE, Medline, Google Scholar, and Scopus. To identify further eligible studies, the reference lists of selected papers and review articles were also scrutinized. Other sources (e.g. papers recommended by experts in the field) were also considered if complied with the eligibility criteria. The search was performed in the period between 22 to 28 November 2021.

### Search strategy

The search strategy was designed in collaboration with an expert librarian. Search terms relatively to technology, nature connectedness, attitudes, and behaviors, as well as various combinations (see an example in Table 2) of these, were used in the database search (Table 2). Given the novelty of the field and the limited extent of literature at the time of the search, no further filters were applied. The search terms were adapted to the specific search rules of the different databases. A detailed description of the search terms for the database search is available in Table A2 of the Appendix.

**Table 2.** Database search algorithms for each main topic.

Main topic	Database search algorithm
#1 Technology #2 Nature #3 Nature connectedness #4 Behavior/behavioral intention to visit nature #5 Attitudes toward the natural world and wildlife	((immersive OR virtual OR mixed OR extended OR simulat* OR high-immersive OR semi-immersive OR “full immersive”) W2 (realit* OR technolog* OR environment* OR screen* OR experience* OR world* OR room* OR device* OR display* OR video* OR scenario*) OR “Cave Automatic Virtual environment” OR CAVE OR “360 degree scenario*” OR “360 degree video*” OR “computer generated” OR “head mounted” OR headset OR “head set” OR CAREN OR “Computed assisted rehabilitation environment”) (natur* OR park OR parks OR forest* OR outdoor* OR bluespace* OR “blue space*” OR greenspace* OR “green space*”) ((connect* OR contact OR relatedness OR involvement OR inclusion OR oneness OR commitment OR relationship*) W2 (natur* OR environment*)) (intent* W2 (visit* OR environment*)) OR behavio?r* OR (t?urism W2 promotion* OR destination* OR marketing OR demand*) (intent* W2 (visit* OR environment*)) OR behavio?r* OR (t?urism W2 promotion* OR destination* OR marketing OR demand*) (destination W1 marketing) OR attitude*)
#1 AND #3	
#1 AND #2 AND #5	
#1 AND #2 AND #4	
#1 AND #2 AND #3	

### Selection and management of relevant papers

The search results were collected and organized in Rayyan (HBKU Research Complex, Doha, Qatar), which was also applied as a tool to exclude duplicates. Two researchers (EB and KS) scrutinized the search outcome by title and abstract, with disagreements being resolved by a third researcher (GC). An independent examination of the full text of potentially eligible papers was conducted by two researchers (EB and GC), with any disagreements being resolved by a third researcher (TEM). The authors of the original papers were reached via email if any clarification was needed. This process was concluded in January 2022.

### Data extraction

The data extraction was performed by two researchers (EB and EP), with quality assurance being provided by a third researcher (GC), and tabulated in Excel sheets (completed in February 2022). As shown in Table 3, the extracted data included: (i) general information about the paper, (ii) characteristics of the sample, (iii) study design, (iv) details of the IVN technology, (v) duration of exposure to the IVN, (vi) details of the control/comparison, (vii) specific nature connectedness measurement, and (viii) brief overview of key findings. When summary statistics or other relevant information was missing in the selected paper, the relative contact authors were reached by e-mail and asked to provide the required information.

**Table 3.** Characteristics of studies.

Reference	Sample	Study design	Intervention	Control	Posture during exposure	Intervention and control exposure time	Instrument
Ahn et al.^46^ (study 1)	University students (n = 54; age: 20.40 ± 1.24 years; gender: 27 females, 22 males), Western United States	RCT, parallel design, post-assessments	Interactive computer-generated simulation of a pasture environment and animals delivered through HMD (n = 25)	Computer-generated simulation of a pasture environment and animals delivered through a screen (n = 24)	Quadrupedal position	*n.a.*	INS^44^
Ahn et al.^46^ (study 2)	University students (n = 54; age: 21.20 ± *n.a.* years; gender: 28 females, 26 males), Western United States	RCT, parallel design, post-assessment with follow up after 1 week	Computer-generated simulation of marine natural environment and wildlife delivered through HMD (n = 31)	Computer-generated simulation of marine natural environment and wildlife delivered through a screen (n = 22)	Upright position	*n.a.*	CNS, trait version^6^
Ahn et al.^46^ (study 3)	University students (n = 126; age: 20.10 ± 2.29 years; gender: 82 females, 44 males), United State	RCT, parallel design, post-assessments	Computer-generated simulation of marine natural environment and wildlife delivered through HMD (n = 84)	Computer-generated simulation of marine natural environment and wildlife delivered through a screen (n = 42)	Upright position	*n.a.*	CNS, trait version ^6^
Chan et al.^47^ (study 1)	Undergraduate students (n = 30; age: 0.50 ± 1.50 years; gender: 21 females, 9 males), Singapore	RCT, crossover design, pre-post assessments	Computer-generated simulation of forest environment delivered through HMD (n = 30)	Interactive computer-generated built environment delivered through HMD (n = 30)	Walking on the spot	5 min	CNS, state version^11^
Chan et al.^47^ (study 2)	Older adults (n = 20; age: 72.70 ± 8.80 years; gender: 18 females, 2 males), Singapore	RCT, crossover design, pre-post assessments	Computer-generated simulation of forest environment delivered through (HMD; n = 20)	Interactive computer-generated built environment delivered through HMD (n = 20)	Sitting on a chair	3 min	Verbal question “How connected to nature do you feel?”
Filter et al.^48^	University students (n = 50; age: 23.76 ± 3.73 years; gender: 36 females, 14 males), Germany	RCT, parallel design, pre-post assessments	360° video of forest environment and wildlife delivered through HMD (n = 25)	Video of forest environment and wildlife delivered through a computer screen (n = 25)	Sitting on a chair	3 min 36 s	Adapted general attitude toward wildlife^49^
Sneed et al.^50^	University students (n = 73; age: 25.70 ± 8.80 years; gender: 44 females, 29 males), United States	RCT, parallel design, pre-post assessments	360° video of a park environment delivered through HMD (n = 27)	Condition 1: 360° video of a library (n = 21) Condition 2: Walk in the same park shown in the IVN condition (n = 25)	Condition 1: Sitting on a chair Condition 2: Walking	12 min	1. NRS^7^ 2. SINS^51^
Soliman et al.^52^	Undergraduate students (n = 227; age: 21.20 ± 6.42 years; gender: 158 females, 67 males, 1 other, 1 undisclosed), Canada	RCT, parallel design, post-assessments	360° video various natural environments and wildlife delivered through HMD (n = 57)	Condition 1: Video of various natural environments and wildlife delivered through a computer screen (n = 58) Condition 2: 360° video of a built environment delivered through HMD (n = 56) Condition 3: Video of a built environment delivered through a computer screen (n = 56)	Sitting on a chair	4 min	INS^44^ and CNS, trait version^6^
Yeo et al.,^53^	Adult volunteers, >18 years (n = 96; age: *n.a.*; gender: 50 females, 46 males), England (UK)	RCT, parallel design, pre-post assessments	Condition 1: 360° video of marine natural environment and wildlife delivered through HMD (n = 31) Condition 2: Interactive computer-generated simulation of marine natural environment and wildlife delivered through HMD (n = 34)	Video of a marine natural environment on a TV screen (n = 31)	Sitting on a chair	5 min	INS^44^, modified as a state measure and adjusted to specific nature content

*Note. n.a.*: not available; CNS: Connectedness with Nature Scale; HMD: head-mounted display; INS: Inclusion of Nature in the Self; NRS: Nature Relatedness Scale; RCT: randomized controlled trial; SINS: State of Independence with Nature Scale.

### Assessment of studies’ risk of bias

The revised risk of bias (RoB2)^54^ for RCTs and crossover trials were used to appraise the risk of bias for the selected studies, as recommended by the Cochrane's guidance for systematic review.^55^ Through signaling questions that feed into an algorithm, the RoB2 tool evaluates the risk of bias associated with (i) the randomization process, (ii) possible deviations from intended interventions, (iii) possible missing outcome data, (iv) measurement of the outcome, and (v) possible selection of reported result (e.g. whether the authors conducted the analyses in accordance with a predetermined plan and/or whether they presented only selected findings). RoB2 for crossover trials^55^ includes an additional domain concerning the time between repeated assessments and possible carryover effects. The risk of bias associated with the individual domains is appraised as “low,” of “some concerns,” or “high.” The overall risk of bias for each study is determined based on the highest bias level among the assessed domains. For more details, please refer to the RoB2 manual (p. 4, Table 1).^56^ The risk-of-bias assessment was independently performed by two authors (EB and VS), with disagreements being resolved through discussion, and completed in May 2022.

### Synthesis methods

Synthesis of findings on pre-post exposure to IVN was done narratively, while quantitative synthesis through meta-analysis was conducted to summarize the effectiveness of IVN versus comparison conditions. According with the Cochrane Consumers and Communication Group guidelines to perform a quantitative synthesis,^57^ a quantitative synthesis of data through meta-analysis was performed when: (i) the outcomes were similar and could be reasonably combined at a conceptual level, (ii) the interventions and control/comparisons conditions were fairly alike, (iii) summary statistics were accessible from the selected papers or furnished by the respective authors, and (iv) at least two of the selected studies possessed all the characteristics outlined at in the previous points.

As by design for this particular systematic review, all the outcomes in the included studies could be reasonably combined. However, the included studies showed substantial differences in relation to the interventions and control/comparison conditions. This was deemed at a possible source of heterogeneity, as supported by high levels of heterogeneity for all studies pulled together (*I^2 ^*= 94%). Hence, the studies were clustered for the type of IVN and comparison condition, resulting in four main groups:

1. *Studies comparing IVN versus non-immersive virtual nature scenarios (six studies, eight outcomes)*
2. *Studies comparing IVN versus immersive virtual built environment (four studies, six outcomes)*
3. *Studies comparing IVN versus non-immersive virtual built environment (one study, two outcomes)*
4. *Studies comparing IVN versus actual nature (one study, two outcomes)*

For each group, a meta-analysis was conducted applying a random-effect model to estimate the overall standardized effect size (Hedge's *g*).^58^ Heterogeneity (*I^2^*) was interpreted consistently with Cochrane's guidelines^57^ as follows: (i) < 40% was deemed as “not important”; (ii) 30%–60% as “moderate”; (iii) 50%–90% as “substantial”; (iv) 75%–100% as “considerable.”

The heterogeneity levels were moderate to considerable in all groups. Thus, according to the Cochrane's guidelines,^57^ possible sources of heterogeneity were explored by evaluating subgroups of studies. From this exploration, it emerged that the type of IVN (360° videos or computer-generated scenarios) and the duration of the exposure (<5 or >5 min) were relevant sources of heterogeneity within groups 1 and 2, respectively. Indeed, clustering the studies in such a way resulted in substantial reductions in the heterogeneity levels. The subgroup analysis was not conducted for groups 3 and 4 because of the insufficient number of eligible studies.

Hence, subgroup analyses were performed by conducting meta-analyses for the following subgroups:

1. *Studies comparing IVN versus non-immersive virtual nature scenarios:*
  - *Studies using 360° video (four outcomes) vs.*
  - *Studies using computer-generated scenarios (four outcomes)*
2. *Studies comparing IVN versus immersive virtual built environment:*
  - *Studies with IVN exposure < 5 min (four outcomes) vs.*
  - *Studies with IVN exposure > 5 min (two outcomes)*

The meta-analyses were conducted in SPSS® Statistics version 28.0.0.0.1 (IBM corp., New York, USA). Statistical significance was assumed with *p* < 0.05. The results were presented through forest plots showing the standardized mean difference, confidence interval of effect size, and overall effect size of the individual studies as well as the overall outcomes scores.

It should be stressed that the meta-analysis and the subgroup analyses were limited by a small number of outcomes—in some cases, leaving only two outcomes from the same study to be included. Even though the analyses were conducted in compliance with the Cochrane's guidelines,^57^ because of the small number of studies, caution is needed when interpreting the results and drawing conclusions based on them.

## Results

### Selected studies

**Figure 1.**
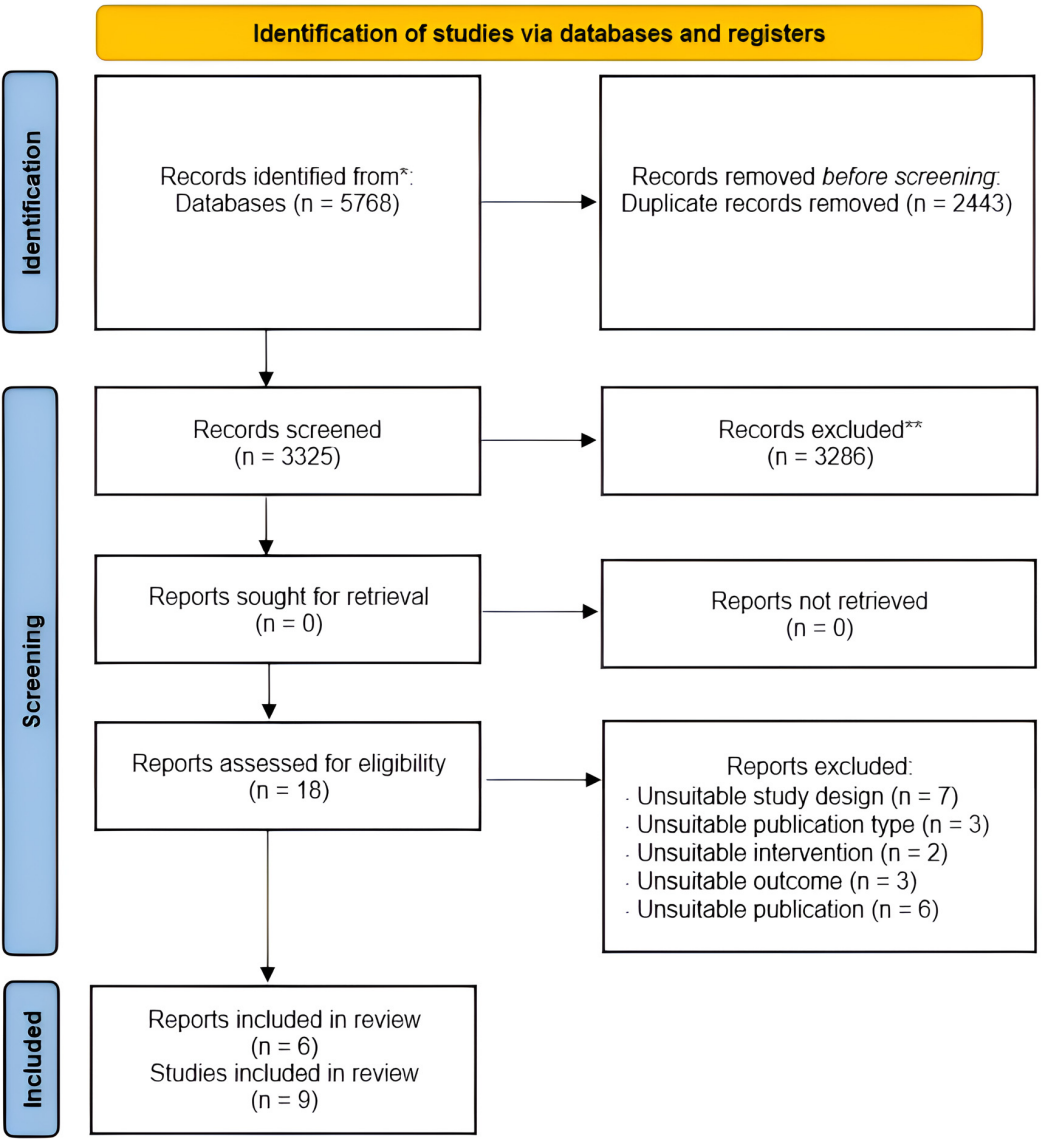
PRISMA flow diagram, adapted from Page et al.^40^

The databases search yielded 5768 records. After removing duplicates, 3325 records were scrutinized for title and abstract. A total of 18 relevant papers were selected for the full-text screening. Of these, six studies met the inclusion criteria,^46–48,50,52,53^ two of which reported several individual trials,^46,47^ for a total of nine eligible studies. The scrutiny of the reference lists did not yield any additional papers. Papers were excluded because of incompatibility with the criteria regarding study design, incorrect outcomes, intervention, and report status. The reasons for exclusion after scrutiny of the full-text are listed in Appendix (Table A3), while the search and selection process results are summarized in Figure 1.

### Study characteristics

Table 3 shows information regarding participants, study designs, exposure time to the IVN, outcomes, and instruments employed in the included papers.

### Trial design characteristics

All included studies used an RCT design, the majority of which utilized a parallel trial design.^46,48,50,52,53^ Two studies^47^ out of nine conducted an RCT with crossover design, with 1 week in-between the exposure to different conditions. Only one study^46^ included a 1-week follow up after a single exposure. Six studies included two conditions,^46–48^ two included three conditions,^50,53^ while one included four conditions.^52^ Five studies assessed the outcomes in both pre- and post-conditions,^47,48,50,53^ while the remaining four studies collected only post-condition assessments.^46,52^ The exposure time to the IVN varied in the different studies, ranging from 3^47^ to 12 min.^46^ Information regarding the duration of the exposure to the IVN was not available in three studies^46^ and was not provided by the original authors after the request.

### Participants

Altogether, the studies included 730 participants, with individual samples ranging from 20^47^ to 227 participants.^52^ All studies included participants of both genders, though there was a predominance of females (n = 306) compared to males (n = 192). The samples consisted of university students in seven studies,^46–48,50,52^ participants recruited from the general public in one study,^47^ and senior citizens in one study.^47^ The mean age of the participants across the studies ranged from 20.10^46^ to 72.70 years.^47^ The studies took place in five different countries: four in the United States,^46,50^ two in Singapore,^47^ one in Germany,^48^ one in England,^47^ and one in Canada.^52^

### Hardware used in the interventions

Concerning the technology adopted in the selected studies, standalone^46,48,51,52^ and high-hand HMD devices^47,53^ were predominantly employed. It is important to highlight key differences in the type of IVN content. In four studies,^48,50,52,53^ the IVN content consisted of a 360° video. Nine studies used a computer-generated scenario for their intervention, of which four^46,47,53^ allowed the users to move and interact with the virtual world, while five were non-interactive.^46,48,50,52^

### IVN content used in the interventions

The visual features of the IVN scenario differed across the included studies, varying from underwater settings to forest environments, with and without the presence of wildlife. A marine environment and corresponding fauna were reproduced in four IVNs conditions,^46,53^ three of which were computer-generated scenarios,^46,53^ and only one 360° video.^47^ Among these, in one computer-generated IVN^47,53^ participants were able to move into the virtual world through the use of a hand-held controller, while in the other ones, participants could only passively observe the environment while embodying a coral avatar—this experience was augmented by a researcher poking them to simulate a fishing net.^46^ The other marine IVN was a 360° video.^47^ Four IVN represented landscapes dominated by trees or foliage plants, specifically forests^47,48^ and wooded trails along a pond.^46^ Among these, two were computer-generated scenarios, in which participants could move in the virtual world through hand-held controllers or by walking on the spot through special trackers attached to their legs via knee pads.^47^ The other two IVNs were 360° videos,^48,50^ which the participants viewed while sitting on a chair. Only one^48^ of these IVNs (a 360° video) included animals, specifically wolfs wandering around the forest and sometimes getting close to the participant's viewpoint. One of the computer-generated IVNs represented a pasture,^46^ in which the participants embodied a cow avatar, moving around the virtual world in a quadrupedal position, with their movements being tracked through LED markers placed on their hands and head. Finally, one IVN^52^ consisted of a 360° video showing several clips representing different types of landscapes (forests, mountains, and rivers) as well as wildlife (i.e. deer).

### Comparison conditions

The comparison conditions consisted of immersive built environments delivered through HMDs in four studies.^47,50,52^ In seven studies, the comparison condition consisted of virtual scenarios delivered through non-immersive displays (i.e. computer screens^46,48,52^ and TV monitor^47^). Six of these studies depicted a natural environment (generally matching the scenario depicted in the IVN condition), while one depicted a built environment (i.e. the inside of a library). In one study, the comparison condition consisted of a visit to an actual naturalistic location, which was the same as depicted in the IVN condition.^46^

### Study outcomes

Details about the specific outcomes and relative instruments employed in the eligible studies can be found in Table 3. The state version of the Connectedness with Nature Scale^11^ was employed in one study,^47^ the trait-version of the same scale was employed in three studies,^46,52^ and the Inclusion of Nature in Self^44^ was adopted in three studies.^46,52,53^ One study^52^ used both the trait-version of the Connectedness with Nature Scale^6^ and the Inclusion of Nature in Self.^44^ Only one study^47^ adapted the original Inclusion of Nature in Self^44^ scale to specify the environment context and to better capture the state dimension of nature connectedness.^53^ One study^46^ used two different instruments to assess nature connectedness, the Nature Relatedness Scale^7^ and the State of Independence with Nature Scale.^51^ In one study,^47^ since many of the participants were illiterate, nature connectedness was assessed by verbally asking the participants how connected to nature they felt at the current moment (1 = very slightly or not at all; 5 = extremely). One study^48^ investigated the attitudes toward wolves using an adapted version of the general attitude toward wildlife assessment.^49^ No included study measured behavioral dimensions of nature connectedness, such as outcomes relative to visits or intention to visit natural locations.

### Risk of bias

**Figure 2.**
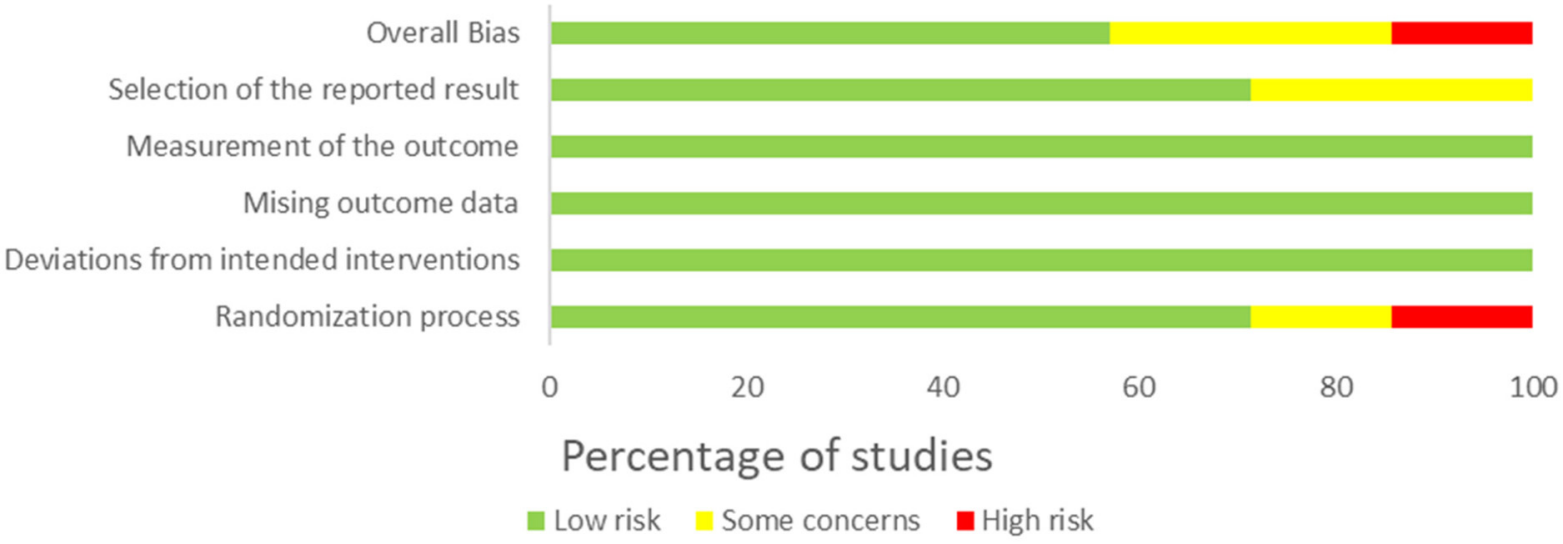
Summary of risk-of-bias judgment for RCT with parallel design.

**Figure 3.**
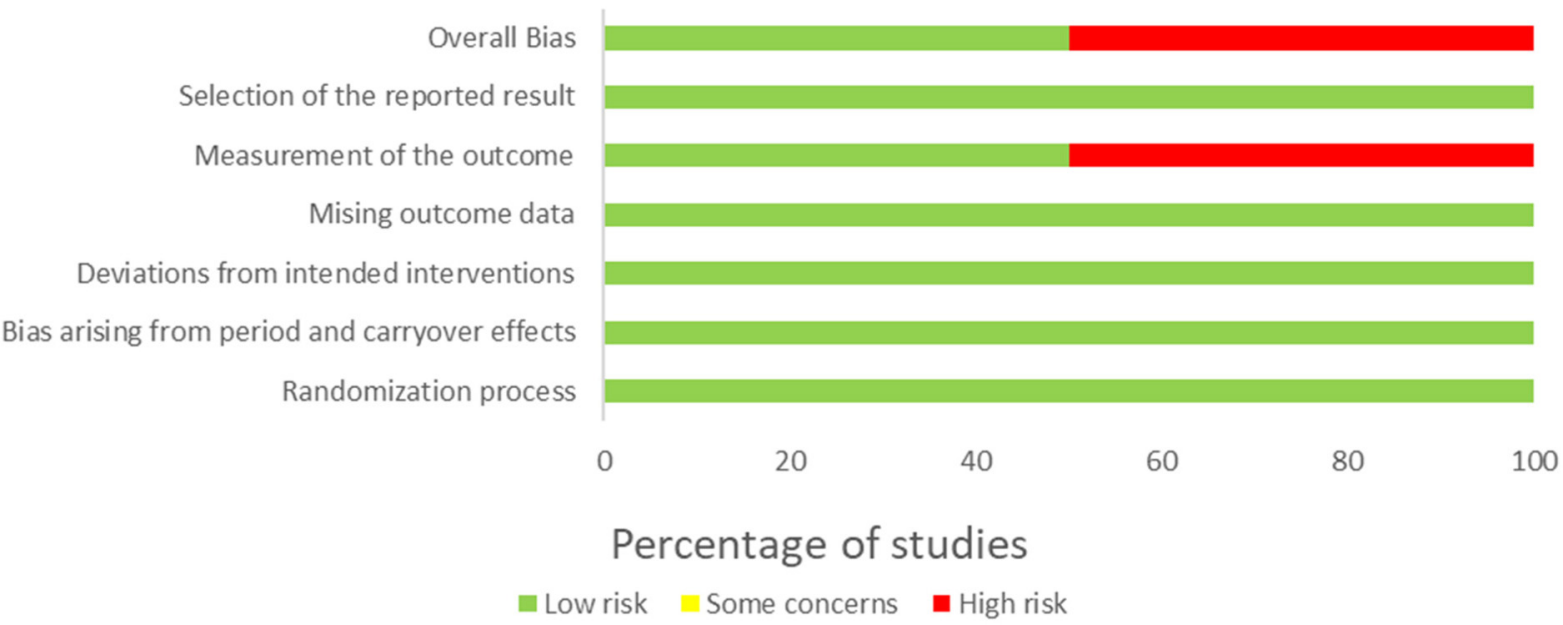
Summary of risk-of-bias judgment for RCT with crossover design.

The summary of the risk-of-bias assessments for the included studies is provided in Figure 2 for the RCTs with a parallel design and Figure 3 for the RCTs with a crossover design. The detailed results for each domain in each included study are displayed in Tables 4 and 5, respectively.

**Table 4.** Risk-of-bias judgment for each RCT with parallel trial design.

Reference	Domain 1	Domain 2	Domain 3	Domain 4	Domain 4	Overall bias
Yeo et al.^53^	Low	Low	Low	Low	Low	Low
Soliman et al.^52^	Some concerns	Low	Low	Low	Low	Some concerns
Sneed et al.^50^	High	Low	Low	Low	Some concerns	High
Filter et al.^48^	Low	Low	Low	Low	Some concerns	Some concerns
Ahn et al.^46^	Low	Low	Low	Low	Low	Low
Ahn et al.^46^	Low	Low	Low	Low	Low	Low
Ahn et al.^46^	Low	Low	Low	Low	Low	Low

*Note.* The table displays the results for each of the five domains of bias, and the overall risk of bias.

Domain 1: randomization process; Domain 2: deviations from intended interventions; Domain 3: missing outcome data; Domain 4: measurement of the outcome; Domain 5: selection of the reported result.

**Table 5.** Risk-of-bias judgment for each RCT with crossover trial design.

Reference	Domain 1	Domain S	Domain 2	Domain 3	Domain 4	Domain 5	Overall bias
Chan et al.^47^ (study 1)	Low	Low	Low	Low	Low	Low	Low
Chan et al.^47^ (study 2)	Low	Low	Low	Low	High	Low	High

*Note*. The table displays the results for each of six domains of bias, and the overall risk of bias.

Domain 1: randomization process; Domain S: bias arising from period and carryover effects; Domain 2: deviations from intended interventions; Domain 3: missing outcome data; Domain 4: measurement of the outcome; Domain 5: selection of the reported result.

### Risk of bias in RCTs with parallel design

Four^46,53^ out of seven studies were considered as having *low* risk of bias, while one trial^46^ was evaluated as having a *high* risk of bias due to low sample homogeneity. Two studies^48,52^ reported *some concerns* due to missing information^52^ or inadequate randomization and selection of the reported results domains.^48^

### Risk of bias in RCTs with crossover design

Two studies applied a crossover design.^47^ Due to the lack of information regarding the randomization process and selection of the reported results, the critical appraisal for one study was deemed as *some concerns.* The other study was evaluated as having a *high* risk of bias, mainly due to the measurement of the outcome, as the data were verbally collected through a not validate instrument.

## Synthetis of findings

### Pre- versus post-exposure assessments (narrative synthesis)

A total of five studies^47,48,50,53^ analyzed nature connectedness pre- and post-exposure, and four of which^47,50^ found a statistically significant increment of nature connectedness levels in the IVN conditions. Two of the studies were assessed as low risk of bias,^47,53^ one as some concerns,^48^ and two as high risk of bias.^47,50^ Only one study^48^ found no significant increments in the levels of nature connectedness measured pre- and post-exposure to the IVN—to be noted that this was the study that measured attitudes toward wildlife. The only study that collected a follow-up assessment of nature connectedness 1 week after the exposure,^46^ found that the heightened levels of nature connectedness experienced by the participants immediately after the IVN exposure weakened by the time of the follow-up assessment.

### Studies comparing IVN versus non-immersive virtual nature

**Figure 4.**
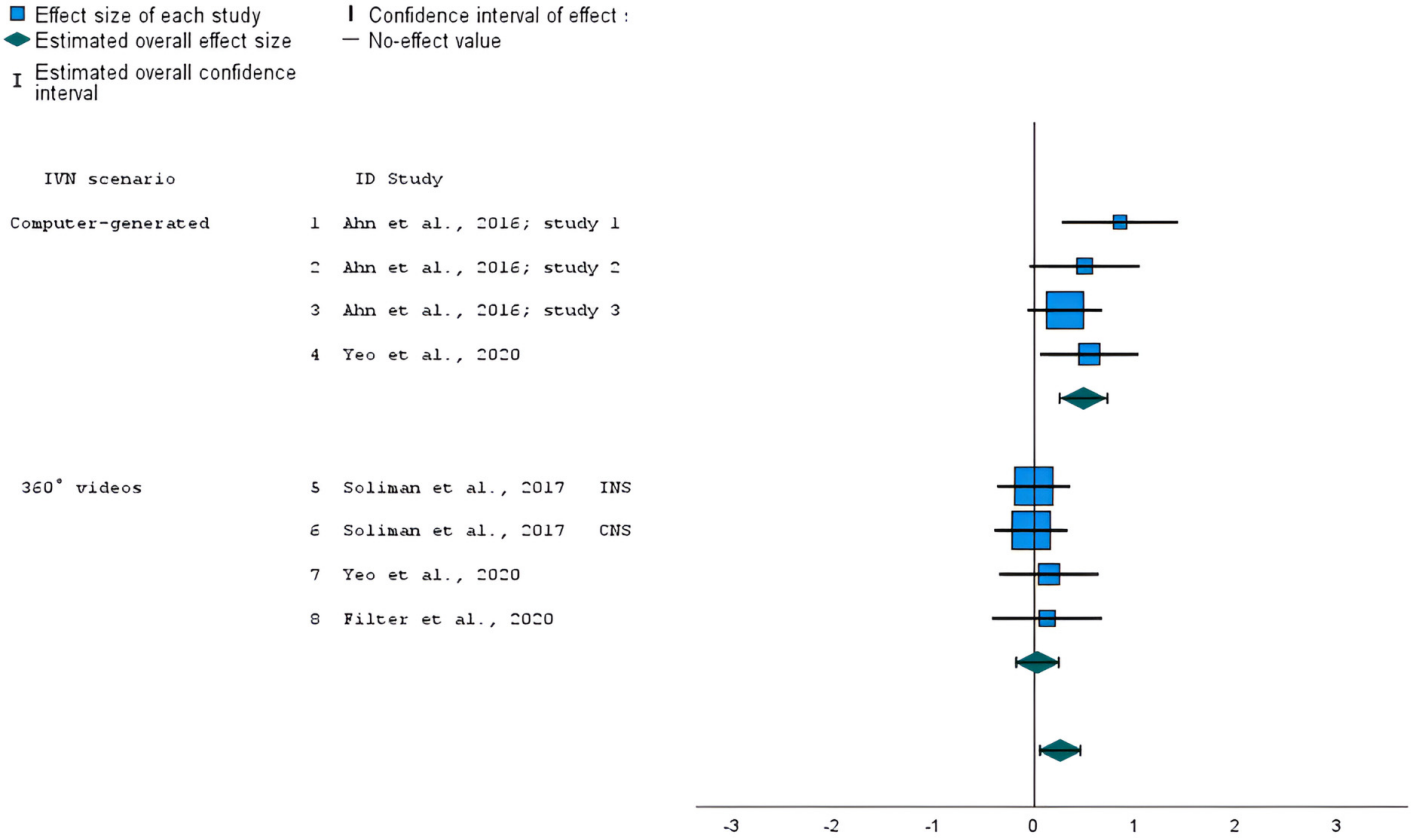
Forest plot showing the standardized mean difference of nature connectedness scores in studies that compared 360° video and computer-generated IVNs versus non-immersive virtual nature. The overall effect size was estimated using a random-effects model. INS: Inclusion of Nature in the Self; CNS: Connectedness with Nature Scale.

Six^46,48,52,53^ out of nine studies (eight outcomes in total) compared IVN versus non-immersive virtual nature (e.g. videos or computer-generated scenarios displayed on a two-dimensional screen). Four of the studies were assessed as low risk of bias^46,53^ and two had some concerns.^48,52^ The meta-analysis of these studies showed a statistically significant overall effect on nature connectedness in favor of the IVN condition (*g* = 0.26, *z* = 2.53, *95% confidence interval (CI)* = 0.06–0.45, *p* = 0.011, Figure 4), with not important to moderate levels of heterogeneity (*I^2^* = 35%). Even though the heterogeneity for this group was relatively low, a subgroup analysis was conducted dividing the studies based on the type of IVN (computer-generated scenarios or 360° videos) to investigate possible heterogeneity sources. The subgroup analysis on the four outcomes that used a computer-generated IVN showed a statistically significant effect on nature connectedness scores in favor of IVN, compared with the non-immersive virtual nature condition (*g* = 0.49, *z* = 4.04, *95% CI* = 0.25–0.72, *p* < 0.001; Figure 4), with not important levels of heterogeneity (*I^2 ^*< 0.5%). On the other hand, the subgroup analysis for the four outcomes that used 360° videos in the IVN conditions showed no statistically significant overall effect (*g* = 0.03; *z* = 0.30, *95% CI* = −0.18 to 0.24, *p* = 0.766; Figure 4), with important levels of heterogeneity (*I^2 ^*< 0.5%).

### Studies comparing IVN versus immersive virtual built environments

**Figure 5.**
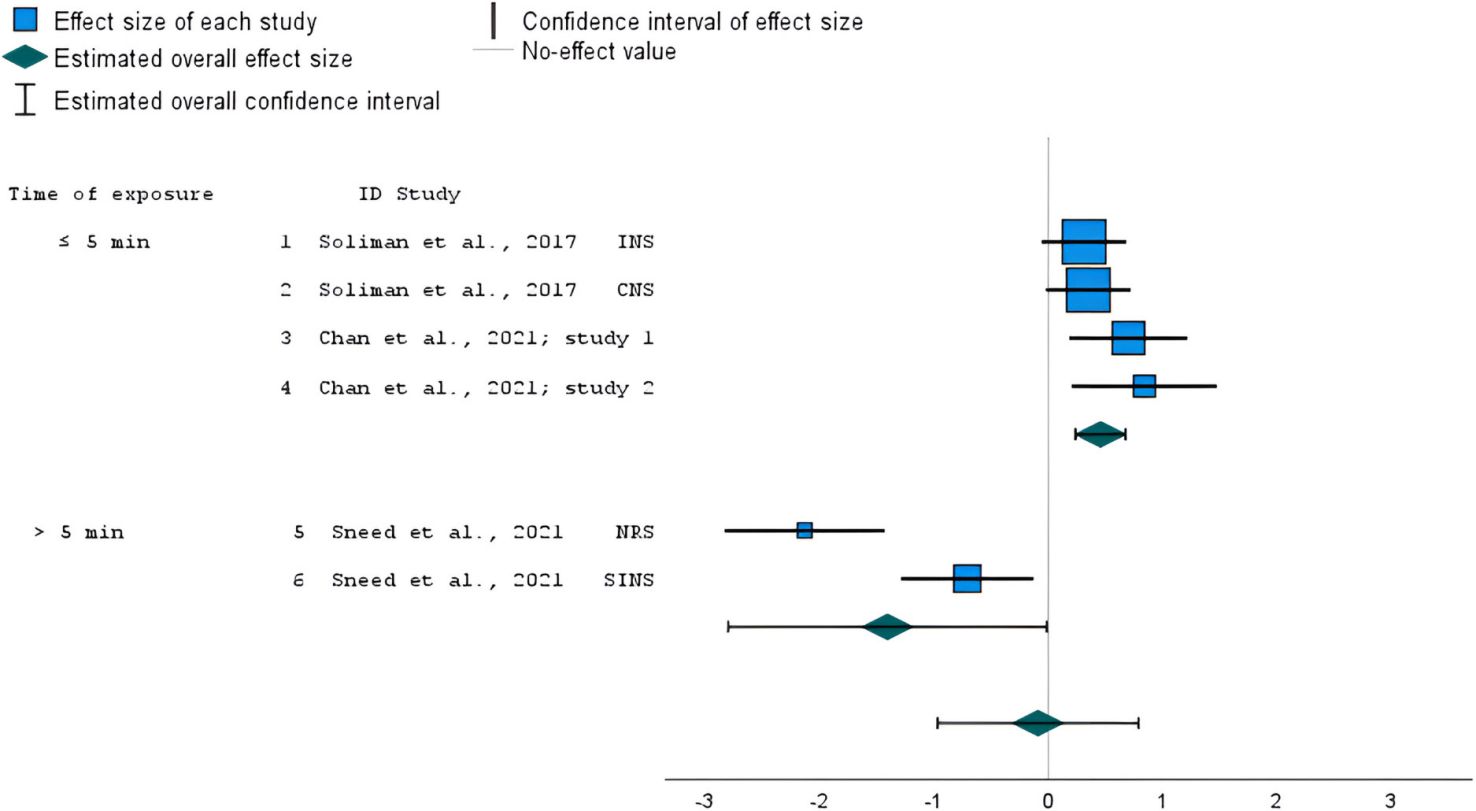
Forest plot showing the standardized mean difference of nature connectedness scores in studies that compared IVN versus immersive virtual urban environment with shorter (≤5 min) and longer (12 min) exposure. The overall effect size was estimated using a random-effects model. INS: Inclusion of Nature in the Self; CNS: Connectedness with Nature Scale; NRS: Nature Relatedness Scale; SINS: State of Independence with Nature Scale.

Four of the included studies,^47,50,52^ which included six outcomes in total, compared IVN versus immersive virtual built environments. One of the studies were assessed as low risk of bias,^47^ one as some concerns,^52^ and two as high risk of bias.^47,50^ The meta-analysis of these studies found no statistically significant overall effect (*g* = −0.09, *z* = −0.20, *95% CI* = −0.97–0.79, *p* = 0.839), with a considerable level of heterogeneity (*I^2 ^*= 95%). Due to the considerable levels of heterogeneity, a subgroup analysis was performed dividing the studies based on the duration of the exposure (≤5 or >5 min). The subgroup analysis conducted over the three outcomes that employed a shorter exposure (≤5 min)^47,52^ showed a statistically significant effect on nature connectedness scores in favor of IVN (*g* = 0.46, *z* = 4.09, *95% CI* = 0.27–0.67, *p* < 0.001; Figure 5), with not important levels of heterogeneity (*I^2 ^*< 0.05%). Quite the opposite, the subgroup analysis of the outcomes (both assessed in the same study) that used a longer exposure (>5 min) showed a marginally significant effect on nature connectedness scores in favor of the comparison condition (*g* = −1.41, z = −1.98, *95% CI* = 2.80 to −0.01, *p* = 0.048; Figure 5), with considerable levels of heterogeneity (*I^2 ^*= 89%).

### Studies comparing IVN versus non-immersive virtual built environments

**Figure 6.**
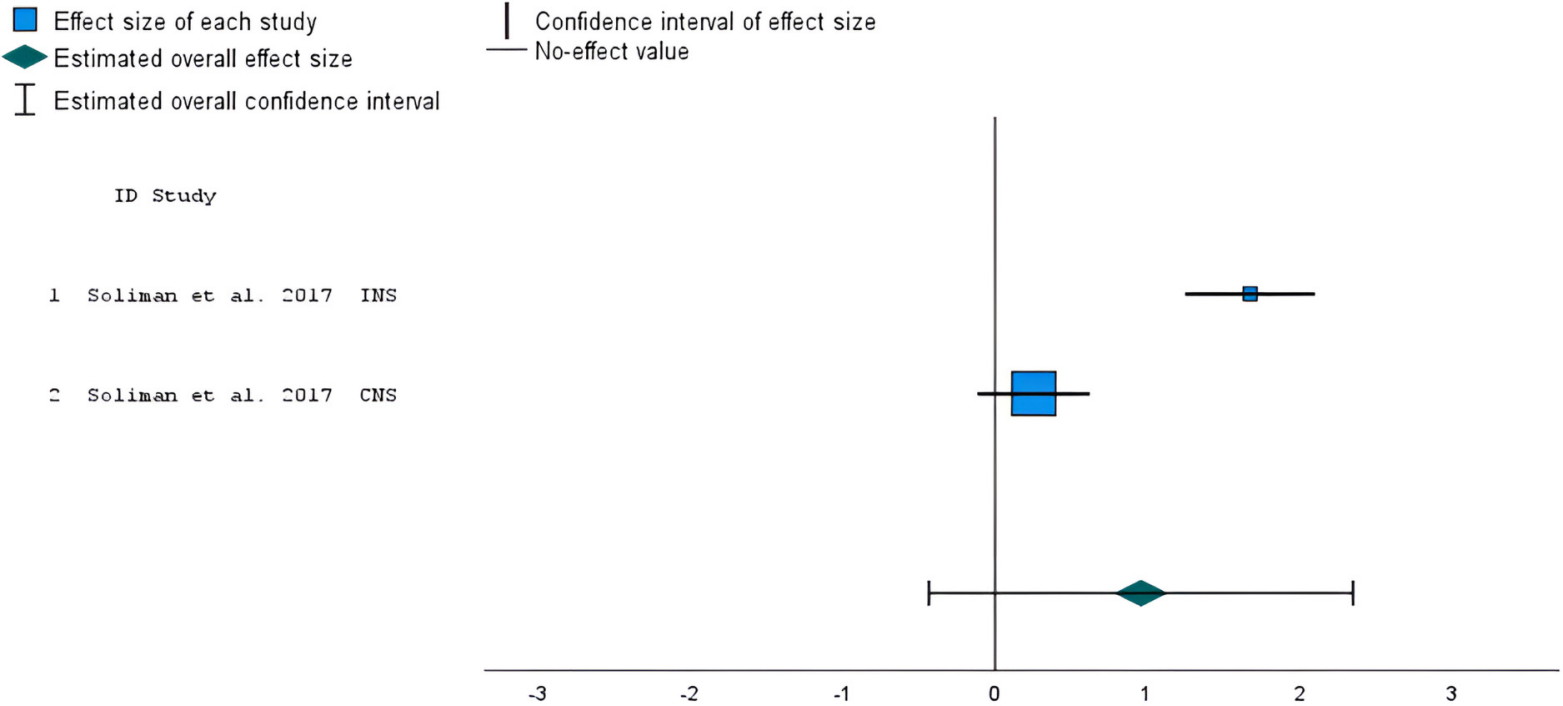
Forest plot showing the standardized mean difference of nature connectedness scores in studies that compared IVN versus non-immersive virtual built environment. The overall effect size was estimated using a random-effects model. INS: Inclusion of Nature in the Self; CNS: Connectedness with Nature Scale.

Only two outcomes, collected in the same study,^52^ were available for meta-analysis comparing the effects of IVN versus a non-immersive virtual build environment on nature connectedness. The study's risk-of-bias assessment resulted as having some concerns. The meta-analysis showed no statistically significant overall effect (*g* = 0.96, *z* = 1.35, *95% CI* = −0.43 to 2.35, *p* = 0.177; Figure 6), with considerable levels of heterogeneity (*I^2 ^*= 96%).

### Studies comparing IVN versus actual nature experiences

**Figure 7.**
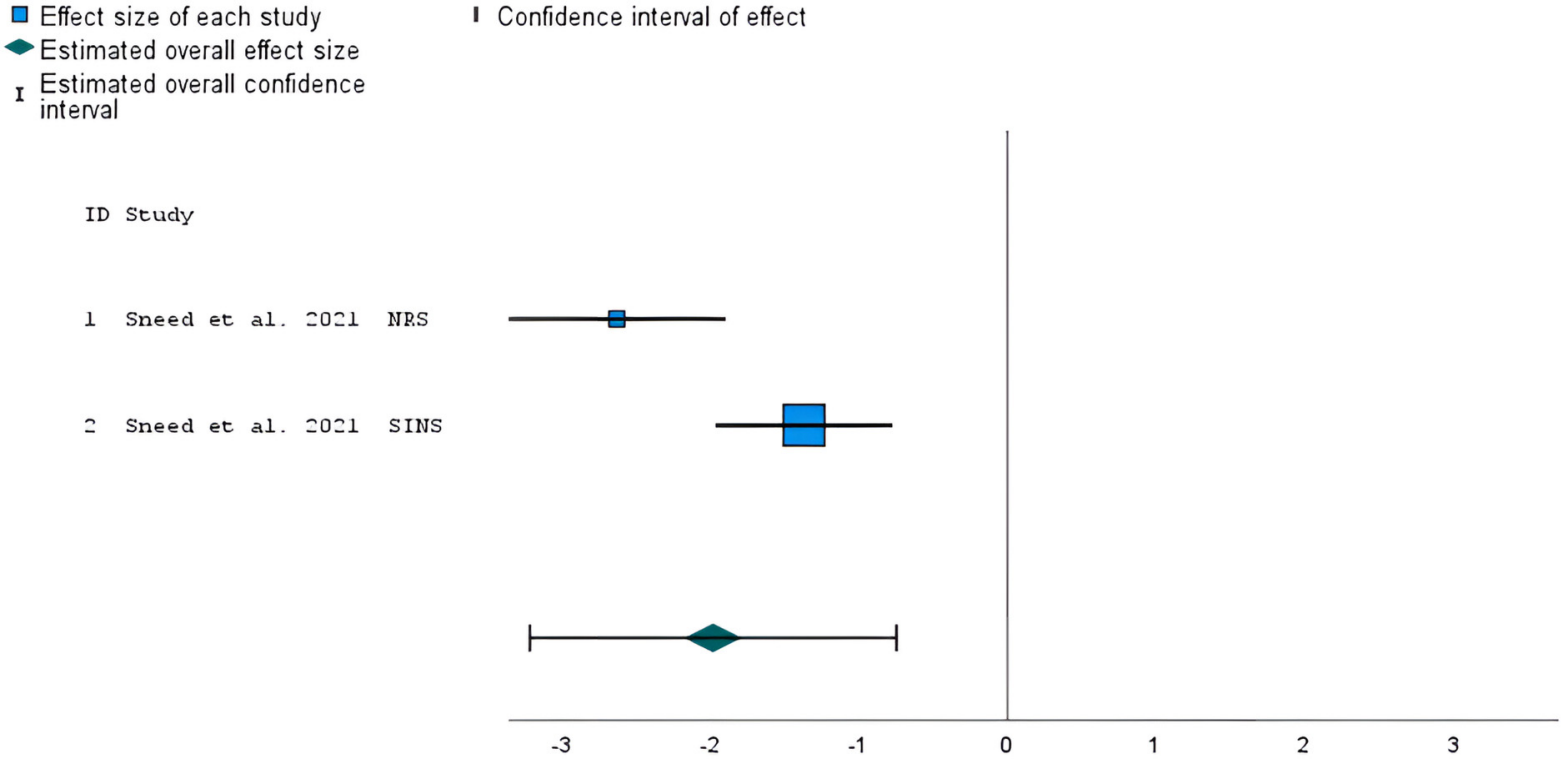
Forest plot showing the standardized mean difference of nature connectedness scores in a study that compared IVN versus an actual nature experience. The overall effect size was estimated using a random-effects model. NRS: Nature Relatedness Scale; SINS: State of Independence with Nature Scale.

Only two outcomes, collected in the same study,^46^ were available for meta-analysis comparing IVN with an actual nature experience. The study was assessed as high risk of bias. The meta-analysis showed a statistically significant effect on nature connectedness scores in favor of actual nature experiences (*g* = −1.98, *z* = 1.35, *95% CI *= −3.21 to −0.75, *p* < 0.001; Figure 7), though with considerable levels of heterogeneity (*I^2 ^*= 85%).

## Discussion

### Synthesis of results

Given the positive associations of nature connectedness with both human wellbeing^3^ and actual human–nature interactions,^2^ and the increasing interest in using IVN as a tool in the promotion of this psychological construct, the aim of this systematic review and meta-analysis was to summarize the effect of IVN on nature connectedness in the general population. A broad concept of nature connectedness as an individual's cognitive, affective, and behavioral connection with the natural world (vegetation, landscapes, and wildlife), assessed through either validated or not validated instruments, was employed.

All but one study that performed pre- and post-exposure assessments of nature connectedness found that IVN elicited statistically significant acute increments of nature connectedness. However, as indicated by the only study that collected a follow-up assessment 1 week after the IVN exposure,^46^ such acute effects appear to be short lived. The quantitative synthesis through meta-analyses indicates a statistically significant effect for the group of studies comparing IVN versus non-immersive virtual nature. However, a further subgroup analysis suggests a differential effect based on the type of technology employed to generate the scenarios with a significant overall effect in favor of IVN for the studies that employed computer-generated IVNs, but not for the studies that used employed 360° video. The meta-analysis for the group of studies comparing IVN versus immersive virtual build environments, found no consistent effects. However, a further subgroup analysis for this group of studies suggests a differential effect based on the duration of exposure, with a significant effect in favor of IVN for the studies that employed a shorter exposure (≤5 min). Further, a marginally significant effect in favor of the immersive virtual built environment conditions was found for the studies with a longer exposure (>5 min). No significant effect was found for the group of studies comparing IVN versus non-immersive virtual built environments, while a significant effect was found for the group of studies comparing IVN versus actual nature experiences, in favor of the latter. Although the findings of this review are limited by the small number of studies available (in some cases, only two outcomes from the same study could be included in the analyses), the findings provide valuable insights into the potential of IVN as a tool in the promotion of nature connectedness, as well as the status of this research field and the specific need for future research.

### Impact of the technological substrate

The findings indicate that IVN tend to elicit increased levels of nature connectedness to a greater extent than non-immersive virtual nature such as images of nature displayed on a computer screens. However, the analysis indicates that a differential effect may exist relatively to the specific technology used to develop the IVN scenarios (computer-generated scenarios or 360° videos). In particular, the findings show that while exposure to computer-generated IVNs tends to have a consistent and positive impact on nature connectedness, exposure to 360° videos appears to have less consistent effects. To the best of the authors’ knowledge, only one study^53^ directly compared the impact of these two specific types of IVN scenarios on nature connectedness, indicating a superior effect for the computer-generated content compared to the matching 360° video. These findings are consistent with previous research suggesting that, in general, IVNs displaying computer-generated scenarios tend to elicit more positive psychological responses and enjoyment than 360° videos.^28,59^ Two main key factors may contribute to explaining this phenomenon: cybersickness and presence. Given its correlation with negative affective responses,^60^ cybersickness is considered to be an important outcome and confounder for IVN research. A recent study showed no significant difference in cybersickness when directly comparing IVN developed as computer-generated or 360° videos.^31^ Still, it is important to consider that the type of technology, the quality of the virtual scenario, and individual characteristics play an important role in enhancing levels of cybersickness,^60^ which may be reflected in the way specific IVNs can elicit affective responses and, possibly, enhanced nature connectedness. Despite the relevance of cybersickness in the field, none of the included studies in this review specifically measured or controlled this outcome. On the other hand, the majority of the included studies in this systematic review collected measures of presence,^46,48,52,53^ intended as an individual's feeling of “being in the virtual environment,”^39^ which is believed to be highly related to cybersickness.^61^ As highlighted in a narrative review by Rebenitsch and Owen^62^, the characteristic of presence may influence the user's experience and correlate with several confounders in studies involving IVN. In general, presence is highly related to the effectiveness of immersive virtual experience, explaining possible variations in the way different IVNs elicit psychological and physiological outcomes.^63–65^ In this respect, greater levels of interactivity tend to be associated with stronger feelings of presence, and since all the computer-generated scenarios included in the subgroup analysis had a certain degree of interactivity, this might have contributed to enhancing the quality of experience and engagement with IVN, explaining the more consistent effects on the participants’ nature connectedness.

### Duration of exposures

The duration of the IVN exposure varied prominently across the studies included in this review. While studies have investigated the impact of the duration of nature exposure on different health and wellbeing outcomes,^66,67^ less is known regarding the most effective duration of exposure to IVN. In the present review, the duration of exposure emerged as a relevant source of heterogeneity in one of the groups of studies comparing IVN and immersive virtual build environments. The outcomes of the subsequent subgroup analysis indicate that a shorter exposure to IVN (≤5 min) may be more effective in eliciting nature connectedness than longer exposures. Although those findings appear to be paradoxical, they are in line with previous research on exposure to actual nature showing that shorter exposure to nature (5 min) had a better effect than longer exposure (<1 h) on other psychological outcomes such as self-esteem and mood.^66^ Specifically with respect to IVN, a narrative review,^31^ highlights the importance of considering the increased risk of cybersickness with increasing exposure time,^68^ hence recommending exposures of about 5 to 10 min in order to gain optimal psychological and biological benefits. It should be noted, however, that only one study^46^ including two outcomes, with a duration >5 min was identified for this study, and the analysis for this subgroup showed considerable levels of heterogeneity, which largely limits the validity of the finding.

### Virtual nature doesn’t measure up to actual nature

In this systematic review, only one study^46^ which included two outcomes, compared the extent to which IVN versus actual nature conditions affected nature connectedness, finding a consistent effect in favor of the latter. Due to few number of outcomes included and the high levels of heterogeneity that emerged from the meta-analysis, these findings need to be carefully interpreted. The results from this qualitative synthesis reveal to be in contrast with a recent review by Sheffield et al.,^20^ which found no differential effects in the extent to which direct (i.e. actual visit of the natural environment) and indirect (i.e. interactions mediated by analog or digital media, including IVN) experiences of nature elicit increased nature connectedness levels. It should be noted, however, that some differences in the methodology and characteristics of the studies in the review by Sheffield et al.^20^ may contribute to explaining this incongruence. Firstly, it should be considered that their meta-analyses were conducted focusing on pre–post and pre-follow-up comparisons, while in the present review (in line with Cochrane's guidance for systematic review^55^) the meta-analyses were conducted only focusing on between-condition comparisons. Moreover, and importantly, Sheffield et al.^20^ included long-term interventions with repeated exposures over several weeks, as opposed to the present review that included only single-exposure interventions. Beyond this, as interactions with actual nature provide a wider and more nuanced range of sensorial experiences and psychophysiological responses, it is reasonable to assume that actual nature experiences support the formation of stronger feelings of nature connectedness, compared to exposure to IVN, as hypothesized by Litleskare et al.^28^ In this respect, considering also the findings indicating that IVN can elicit increased nature connectedness compared with nature exposure mediated by non-immersive technology, the findings of this review are in line with a conceptual framework proposed by Litleskare et al.,^28^ according which individuals’ psychological responses (including enhanced nature connectedness) associated with digitally mediated experience of nature are strongly dependent on the fidelity with which they resample actual nature experiences. IVN experience providing a larger variety of multisensory input beyond the visual (e.g. sounds, smells, haptic sensation, and thermoception) have been proposed to elicit more favorable psychological responses than those that only allowed visual exposure.^69^ This emphasizes the importance of actual nature interactions, as well as realistically frames the role of IVN as a means to foster people's nature connectedness: while IVN could be an important tool to facilitate access to nature among certain population groups, it is largely unreasonable to expect that IVN could be complete substitute interactions with actual nature.

### Scarcity of evidence on long-term effects of IVN on nature connectedness

As all the included studies investigated possible acute changes in nature connectedness after single-exposure interventions, the finding of this review highlights the lack of longitudinal studies investigating the long-term effects of interventions with repeated IVN exposures over a prolonged time span. The one included study that performed a follow-up assessment,^46^ suggests that the increased nature connectedness acutely elicited by the IVN exposure weakened rather quickly (within one week) after the intervention. However, it is pertinent to postulate that long-term interventions with repeated exposures may have the potential to elicit sustained heightened nature connectedness over time. Trait levels of nature connectedness can, indeed, modify across the lifespan suggesting that the emotional attachment to the natural world, as well as the beliefs and behaviors associated with it are dynamic.^70,71^ Furthermore, a recent study published after the completion of the data search^72^ indicates that repeated exposure to IVN (a 360° video) can significantly improve the trait level of nature connectedness in individuals with a low affinity for nature. The findings of the recent systematic review by Sheffield et al.,^20^ which included a broader variety of indirect means of nature contact (both, immersive and non-immersive), also provide support to the assumption that longer interventions may elicit long-living changes in nature connectedness. However, to date, there is simply not sufficient evidence to evaluate the potential of IVN in promoting stable heightened trait levels of nature connectedness.

### Strengths and quality of the existing evidence

The risk-of-bias assessment indicates that the quality of evidence was generally high. Sources of strength are related to the randomization process, measurement of the outcomes, and selection of the reported results. All the included studies employed a concealed random allocation sequence that remained undisclosed until participants were assigned and engaged in the interventions.^46–48,50,52,53^ The assessment of nature connectedness was performed through validated measurement tools, only one study^47^ employed a different approach. In order to estimate an appropriate sample size, most of the studies^47,48,50,52,53^ conducted a priori power calculation. Only one study^52^ conducted a data analysis in accordance with a pre-registered protocol. Nevertheless, after reaching the contact authors of the original papers, it was possible to establish that the majority of the studies^46,47,53^ performed the data analysis in line with a prespecified analysis plan.

Firstly, although state measures of nature connectedness are generally recommended to investigate the specific momentary changes of nature connectedness in relation to short-term interventions, only three studies collected state measures of nature connectedness.^47,50,53^ Although previous literature suggests that trait and state nature connectedness scores are highly correlated,^11^ the use of trait measurements in the context of short-term assessments may limit the instrument’s capacity to detect acute changes. Another possible limitation of the included studies regards the lack of control for possible relevant confounders related to the use of IVN, such as cybersickness, presence, and baseline levels of nature connectedness. Regarding the latter, a recent study specifically selected participants with low baseline levels of nature connectedness,^72^ reporting significantly higher levels of nature connectedness and motivation for engaging with nature after repeated IVN interventions delivered as a 360° video.

### Strengths and limitations

An important strength of this review lies in its methodological approach. Established procedures for conducting systematic reviews were followed, with clear eligibility criteria and a search strategy being preliminarily outlined in a prepublished protocol.^39^ The findings were transparently presented in line with the PRISMA guidelines. The selection of relevant papers and data extraction was independently performed by two authors, with a third one addressing possible disagreement. The risk of bias in the eligible studies was evaluated by two authors through a standardized tool, with any disagreement settled by a third one. A quantitative synthesis of the data through meta-analyses was conducted in line with the Cochrane handbook and guidelines.^55^ Furthermore, most of the authors have previous experience conducting systematic reviews. The University of South-Eastern Norway and its library provided essential support and assistance regarding the literature search. Altogether, this contributed to reducing the degree of subjectivity and possible risk of bias in the research process. Another strength of the present study is its novelty and contribution to a rapidly growing field. Besides providing a systematic synthesis of the currently available evidence on the effects of IVN on nature connectedness, the findings provide valuable knowledge regarding the ongoing debate on the extent to which nature connectedness is a modifiable psychological state.

At the same time, some weaknesses need to be highlighted. Despite the decision of excluding papers not written in English, as well as gray literature, have been taken in order to provide high-quality evidence on the topic, given the limited number of included studies, it may have limited the extent of evidence that could be reviewed and led to studies being overlooked. Potentially relevant papers might not have been identified because of the wide range of search terms in the matter of IVN and nature connectedness. Despite this review was conducted operationalizing the concept of nature connectedness in a broad perspective, several definitions are employed in different research fields. It may represent a limitation that should be dealt with in future research. The database search was concluded in November 2021. Hence, the present review does not include some of the most recent literature that was published after the completion of the data search.^34,72–74^ While these papers meet our inclusion criteria, it should be noted that, because of the characteristics of their design and their outcomes, only one^72^ among these studies (study 2 in Leung et al.^72^) could have been included in our meta-analysis comparing IVN versus immersive urban environments. All other studies could have been only included in the narrative synthesis, as they provide only pre–post assessments^72,73^ otherwise their comparison condition or outcome could not be meaningfully combined with any of the other studies.^34,74^ Nevertheless, this represents a further limitation of the present study that is particularly worth noting. The substantial levels of heterogeneity that emerged from the meta-analysis and subgroup analysis represent a further limitation. This is remarkable, especially considering that the measures of nature connectedness for these meta-analyses were collected among the same participants and within the same study. Possible sources of the unexplained heterogeneity in this review may relate to the use of different scales to measure nature connectedness and to the participants’ baseline levels of nature connectedness. Finally, even though the Cochrane handbook and guidelines state that meta-analyses can be conducted with a minimum of two outcomes,^55^ due to the limited number of eligible studies and the high levels of heterogeneity, the interpretation of the meta-analyses’ results need to be interpreted with caution.

### Implications for future research

In accordance with the findings of the present review, the following recommendations for future research on the effects of IVN on nature connectedness are outlined:

1. More longitudinal studies investigating the possible long-term effects of IVN on nature connectedness are needed. This may include studies investigating the longevity of the acute increases in nature connectedness after single exposures, though follow-up assessments, as well as studies investigating the maintenance of increased nature connectedness through repeated exposures.
2. Studies investigating the effects of IVN on behavioral outcomes related to nature connectedness (e.g. visitation of actual natural locations, engagement with environmental issues, pro-environmental behaviors, etc.), either in the acute or in the long term, are also needed.
3. Future studies should include assessments of possible confounders, and possibly investigate relevant mechanisms that may influence the impact of IVN exposure on nature connectedness. These may primarily include assessments of cybersickness, presence, and baseline level of nature connectedness.
4. Future studies should employ trait or state assessments of nature connectedness as most appropriate for their research question. In particular, it is recommended that researchers performing RCTs with pre–post assessments in concomitance of single-exposures should use state measures of nature connectedness, while trait measurements of nature connectedness could be most appropriate when assessing baseline levels of nature connectedness or long-term effects of single or multiple IVN exposures.

## Conclusion

This systematic review and meta-analysis synthesized the evidence on the effects of IVN on nature connectedness, a topic that increasingly gained interest in several research fields. The results only partially support the effectiveness of IVN in enhancing nature connectedness among the general population. The effectiveness of IVN as a tool in the promotion of nature connectedness may be related to the specific design of such interventions. Factors such as the technological substrate and the duration of the exposure emerge as components that may possibly impact the extent to which IVN can increase nature connectedness. Future research is required into ways of better understanding the acute and long-term effects of IVN, as well as how to optimize the desired effects of this technology.

## Other information

This systematic review was registered in the international prospective register of systematic review (PROSPERO) under the registration number: CRD42021290442. The protocol for this systematic review was published in 2022, https://doi.org/10.1177/20552076221120324.

## Abbreviations

CNS: Connectedness with Nature Scale
HMD: Head-mounted Display
INS: Inclusion of Nature in the Self
IVN: Immersive Virtual Nature
NRS: Nature Relatedness Scale
RCT: Randomized Controlled Trial
SINS: State of Independence with Nature Scale.

## Appendix

**Table A1.** Databases search algorithms for each main topic.

Main topic	Database search algorithm
Technology	((immersive OR virtual OR mixed OR extended OR simulat* OR high-immersive OR semi-immersive OR “full immersive”) W2 (realit* OR technolog* OR environment* OR screen* OR experience* OR world* OR room* OR device* OR display* OR video* OR scenario*) OR “Cave Automatic Virtual environment” OR CAVE OR “360 degree scenario*” OR “360 degree video*” OR “computer generated” OR “head mounted” OR headset OR “head set” OR CAREN OR “Computed assisted rehabilitation environment”)
Nature	(natur* OR park OR parks OR forest* OR outdoor* OR bluespace* OR “blue space*” OR greenspace* OR “green space*”)
Nature connectedness	((connect* OR contact OR relatedness OR involvement OR inclusion OR oneness OR commitment OR relationship*) W2 (natur* OR environment*))
Behavior/behavioral intention to visit nature	(intent* W2 (visit* OR environment*)) OR behavio?r* OR (t?urism W2 promotion* OR destination* OR marketing OR demand*)
Attitudes toward the natural world and wildlife	(destination W1 marketing) OR attitude*)

**Table A2.** Databases search strategy and algorithms.

Main topic	Database search algorithm
Technology AND Nature connectedness	((immersive OR virtual OR mixed OR extended OR simulat* OR high-immersive OR semi-immersive OR “full immersive”) W2 (realit* OR technolog* OR environment* OR screen* OR experience* OR world* OR room* OR device* OR display* OR video* OR scenario*) OR “Cave Automatic Virtual environment” OR CAVE OR “360 degree scenario*” OR “360 degree video*” OR “computer generated” OR “head mounted” OR headset OR “head set” OR CAREN OR “Computed assisted rehabilitation environment”) AND ((connect* OR contact OR relatedness OR involvement OR inclusion OR oneness OR commitment OR relationship*) W2 (natur* OR environment*))
Technological nature AND Attitudes	((immersive OR virtual OR mixed OR extended OR simulat* OR high-immersive OR semi-immersive OR “full immersive”) W2 (realit* OR technolog* OR environment* OR screen* OR experience* OR world* OR room* OR device* OR display* OR video* OR scenario*) OR “Cave Automatic Virtual environment” OR CAVE OR “360 degree scenario*” OR “360 degree video*” OR “computer generated” OR “head mounted” OR headset OR “head set” OR CAREN OR “Computed assisted rehabilitation environment”) AND (natur* OR park OR parks OR forest* OR outdoor* OR bluespace* OR “blue space*” OR greenspace* OR “green space*”) AND (destination W1 marketing) OR attitude*)
Technological nature AND Behavior/behavioral intention to visit nature	((immersive OR virtual OR mixed OR extended OR simulat* OR high-immersive OR semi-immersive OR “full immersive”) W2 (realit* OR technolog* OR environment* OR screen* OR experience* OR world* OR room* OR device* OR display* OR video* OR scenario*) OR “Cave Automatic Virtual environment” OR CAVE OR “360 degree scenario*” OR “360 degree video*” OR “computer generated” OR “head mounted” OR headset OR “head set” OR CAREN OR “Computed assisted rehabilitation environment”) AND (natur* OR park OR parks OR forest* OR outdoor* OR bluespace* OR “blue space*” OR greenspace* OR “green space*”) AND (intent* W2 (visit* OR environment*)) OR behavio?r* OR (t?urism W2 promotion* OR destination* OR marketing OR demand*)
Technological nature AND Nature connectedness	((immersive OR virtual OR mixed OR extended OR simulat* OR high-immersive OR semi-immersive OR “full immersive”) W2 (realit* OR technolog* OR environment* OR screen* OR experience* OR world* OR room* OR device* OR display* OR video* OR scenario*) OR “Cave Automatic Virtual environment” OR CAVE OR “360 degree scenario*” OR “360 degree video*” OR “computer generated” OR “head mounted” OR headset OR “head set” OR CAREN OR “Computed assisted rehabilitation environment”) AND (natur* OR park OR parks OR forest* OR outdoor* OR bluespace* OR “blue space*” OR greenspace* OR “green space*”) AND ((connect* OR contact OR relatedness OR involvement OR inclusion OR oneness OR commitment OR relationship*) W2 (natur* OR environment*))

**Table A3.** Characteristics of excluded studies on the base of full-text screening.

Study	Reason for exclusion
Frank^75^	- Conference proceeding - Uncontrolled trials with only post-assessment measures of nature connectedness - A different set of collected outcome
Smith et al.^76^	Conference proceeding
Yu et al.^77^	- Conference proceeding - A different set of collected outcome
Deringer and Hanley^78^	Measures of nature connectedness were collected only as a baseline control variable
Huang et al.^79^	- Uncontrolled trials with only post-assessment measures of nature connectedness - Only use of non-immersive technology
Tussyadiah et al.^80^	Uncontrolled trials with only post-assessment measures of nature connectedness
Vassiljev et al.^81^	Uncontrolled trials with only post-assessment measures of nature connectedness
Schutte et al.^82^	Measures of nature connectedness were collected only as a baseline control variable
Depledge et al.^83^	- Review - Feature article
Tutwiler et al.^84^	Only use of non-immersive technology
Nilsson et al.^85^	- Review - A different set of collected outcome
